# Whole peritoneal radiotherapy in malignant ascites: a hypothesis-generating conceptual review

**DOI:** 10.1515/med-2026-1546

**Published:** 2026-10-12

**Authors:** Mirosław Kiedrowski, Katarzyna Ziemba, Paweł Banatkiewicz, Przemysław Kapała, Ewa Szulecka, Paweł Nurzyński

**Affiliations:** Department of Oncology, The National Medical Institute of the Ministry of the Interior and Administration, Warsaw, Poland; Ewdomed Hospice and Foundation, Konstancin-Jeziorna, Poland; Radiological Diagnostics Center, Department of Radiotherapy, The National Medical Institute of the Ministry of the Interior and Administration, Warsaw, Poland

**Keywords:** malignant ascites, radiotherapy, peritoneal carcinomatosis, palliative care, symptom management

## Abstract

**Introduction:**

Malignant ascites (MA) impairs quality of life and remains difficult to control with symptomatic treatments. We summarize the rationale, uncertainties, and potential role of whole-peritoneal radiotherapy (WPRT) as an investigational palliative strategy for MA. The available evidence is limited, and no prospective or high-quality data currently establish WPRT as an effective treatment for MA.

**Content:**

We conducted a narrative review of MA in the context of palliative radiotherapy, whole-abdominal and whole-peritoneal irradiation, technical feasibility, and treatment-related toxicity. Because no prospective or systematically designed studies have evaluated WPRT using ascites-specific endpoints, indirect evidence was included. An illustrative radiotherapy planning simulation in a model patient was also presented to demonstrate technical considerations.

**Summary:**

Direct evidence supporting WPRT for symptom control in MA is lacking. However, the available literature provides a plausible rationale for this approach. Published literature supports the technical feasibility of large-field abdominal irradiation in selected oncological settings, but does not establish the feasibility, safety, or efficacy of WPRT specifically for MA.

**Outlook:**

WPRT should be regarded as an investigational palliative approach supported by clinical rationale rather than evidence-based practice. WPRT may extend symptom-directed palliative radiotherapy to selected patients with MA, but its value requires prospective evaluation using ascites-specific endpoints.

## Introduction

The peritoneum is a serous membrane originating from the mesoderm and lining the abdominal cavity. It consists of mesothelial cells supported by a basement membrane and subserous tissue containing blood vessels [1]. The peritoneum produces peritoneal fluid (PF), which reduces friction between the organs. Under physiological conditions, the amount of PF does not exceed 150 mL. Ascites develops when fluid production exceeds absorption. The most common causes of ascites include cirrhosis with portal hypertension, hypoalbuminemia, inflammatory or malignant infiltration of the peritoneum, and obstruction of lymphatic drainage in the abdominal cavity. Other causes include heart failure, tuberculosis, pancreatic diseases, idiopathic ascites in hemodialysis patients, chlamydial peritonitis, nephrotic syndrome, protein-losing enteropathy, systemic lupus erythematosus, hypothyroidism, portal vein thrombosis, Budd-Chiari syndrome, filariasis, and Meigs syndrome [2].

Cancer accounts for approximately 10 % of cases of ascites. In this setting, the term malignant ascites (MA) is applied. MA is associated with abdominal distension, pain, impaired organ function, lower-limb swelling, nausea, vomiting, constipation, and hiccups, all of which may impair quality of life [3]. Symptom relief frequently requires repeated large-volume paracenteses or the placement of a tunneled peritoneal catheter, both of which increase healthcare costs [4]. Repeated PF evacuations worsen hypoalbuminemia. Thus, albumin supplementation may benefit patients with post-paracentesis syndrome [5]. Repeated procedures increase the risk of peritoneal adhesions and compartmentation, making subsequent drainage challenging [6].

Causal treatment of MA is the most desirable option. In many patients, however, optimal palliative care becomes the only option [7]. Table 1 presents the practical comparison of palliative strategies for MA.

**Table 1:** Practical comparison of palliative strategies for malignant ascites.

Strategy	Main role	Expected onset of symptom relief	Patient burden	Main limitations
Repeated paracentesis	Rapid symptomatic relief	Immediate	Repeated procedures, frequent visits, protein loss	Temporary effect; frequent recurrence; risk of hypotension, infection, leakage
Tunneled peritoneal catheter	Home-based recurrent drainage	Rapid after placement	Device care, caregiver involvement	Infection, blockage, leakage, protein loss; may be unsuitable in loculated ascites
Diuretics	Selected patients, especially portal-hypertension component	Slow and variable	Low procedural burden	Often limited efficacy in malignant ascites; renal/electrolyte toxicity
Systemic anticancer therapy	Disease control in treatment-sensitive tumors	Delayed	Depends on regimen	Requires adequate performance status; ineffective in refractory disease
Intraperitoneal/targeted approaches	Selected settings or trial-based therapy	Variable	Procedure- and access-related burden	Limited availability; tumor-specific evidence; toxicity and logistical constraints
WPRT	Investigational strategy targeting diffuse peritoneal disease	Unknown	Non-invasive but requires RT planning and treatment delivery	Indirect evidence for ascites control; uncertain dose, toxicity, and patient selection

Despite ongoing development of novel therapies, the most effective currently available relief strategies remain repeated therapeutic paracentesis and peritoneal drainage systems [8]. Diuretics demonstrate limited efficacy in MA. Intraperitoneal catumaxomab has been demonstrated to prolong puncture-free survival compared with paracentesis alone [9]. Similarly, vidutolimod (CMP-001), a Toll-like receptor 9 agonist, may also have palliative potential in peritoneal carcinomatosis associated with gastrointestinal and pancreaticobiliary cancers [10]. None of those, however, has become a standard of care.

PF drainage provides impressive but temporary symptomatic relief. Thus, additional symptom-directed strategies deserve consideration, particularly those applicable across multiple tumor types. Whole peritoneal radiotherapy (WPRT) may represent a potential dedicated palliative option for MA. Targeting neoplastic infiltration of the peritoneum could theoretically reduce malignant exudation, thereby improving quality of life and decreasing the need for other interventions.

We present a narrative review relevant to WPRT in the context of MA. Given the heterogeneous terminology in this field, we define WPRT as external beam radiotherapy targeting the peritoneal compartment involved by macroscopic or microscopic tumor involvement, with palliative intent in MA. It is distinct from historical whole-abdominal radiotherapy, variably defined whole-abdominal/peritoneal radiotherapy, and intraperitoneal radionuclide therapy, which differ in target concept, delivery, dosimetry, toxicity, logistics, and evidence base. Throughout this review, WPRT refers to a conceptual external-beam approach to peritoneal compartment irradiation for MA-related palliation.

Our work builds on a concept previously outlined in a Letter to the Editor published recently in the Journal of Palliative Medicine [11] and substantially expands on the information and concept presented there, with particular emphasis on the biological rationale, indirect clinical evidence, technical feasibility, safety considerations, and current knowledge gaps. Because no studies were specifically designed to evaluate WPRT as a dedicated treatment for MA, we also discussed indirect evidence from studies on whole-abdominal or abdominopelvic radiotherapy in malignancies with peritoneal involvement. Therefore, this review should be interpreted as a hypothesis-generating conceptual synthesis intended to define the rationale, uncertainties, safety considerations, and research framework for future prospective studies.

## Malignant ascites: pathophysiology, clinical burden, and current palliative management

The pathophysiology of MA is multifactorial. Important mechanisms include neoplastic infiltration of the peritoneum, increased vascular permeability, lymphatic obstruction, impaired fluid resorption, portal hypertension, hypoalbuminemia, and inflammation. In many patients, several of these processes coexist. Peritoneal carcinomatosis is a frequent contributor, particularly in advanced ovarian, colorectal, pancreatic, breast, and gastric malignancies, as well as in selected lung cancers, lymphomas, and peritoneal mesothelioma. In addition to abdominal distension and discomfort, patients may develop dyspnea due to diaphragmatic elevation, early satiety with reduced oral intake, constipation, impaired mobility, edema, fatigue, and progressive nutritional decline. Recurrent drainage procedures, although often inevitable, may deepen hypoalbuminemia and contribute to cachexia. The need for repeated hospital visits or invasive procedures also places an additional burden on patients and caregivers [3], [4], [5], [6], [7], [8].

Repeated paracenteses provide rapid relief but do not prevent fluid reaccumulation. Tunneled peritoneal catheters are efficient, but still associated with device-related issues [4], 8]. Diuretics are generally less effective than in portal hypertensive ascites. Intraperitoneal immunotherapeutic or biologically targeted approaches are promising but remain non-standard [9], 10]. Consequently, novel strategies that provide symptom control and are feasible in routine palliative practice are highly desirable.

## Biological and clinical rationale for radiotherapy in malignant ascites

A rationale for WPRT in MA arises from the role of peritoneal tumor involvement in generating cancer-related exudation. If radiotherapy were able to reduce the number of viable tumor cells within the peritoneum or limit their biological activity, it could potentially reduce fluid production. Such an effect could reduce the need for drainage, lessen symptom burden, and possibly stabilize the nutritional status.

This concept is biologically plausible but remains unproven in the specific setting of MA. The likelihood of benefit would probably depend on the radiosensitivity of the underlying malignancy, the pattern and extent of peritoneal involvement, previous treatments, and the patient’s fitness. Over recent decades, numerous determinants of radioresistance have been identified, including tumor heterogeneity, molecular characteristics, microenvironmental factors, and the role of cancer stem cells and mitochondrial function [12], 13]. Despite intertumoral differences in radiosensitivity, it cannot be excluded that a limited radiotherapeutic effect, such as reduced malignant exudation, could be achieved using a tumor-agnostic approach, analogous to the use of radiotherapy for pain relief or bleeding control. Still, this concept should not be interpreted as implying comparable efficacy across tumor histologies, as the likelihood and durability of response are expected to depend on the intrinsic radiosensitivity of the underlying malignancy. Future studies should therefore stratify outcomes by primary tumor type. To frame this issue within the broader clinical context, Table 2 summarizes the expected relevance of tumor histology and radiosensitivity for the potential use of WPRT in MA.

**Table 2:** Expected relevance of tumor histology and radiosensitivity for WPRT in malignant ascites.

Tumor histology/clinical setting	General radiosensitivity	Expected relevance for WPRT in MA/Implication for future studies
Haematological malignancies with peritoneal involvement, e.g. lymphoma	High	Potentially high probability of biological response, if peritoneal disease is a major driver of ascites. May be considered favorable candidates, provided clinical condition and prognosis allow WPRT.
Ovarian cancer	Moderate to high	Indirect reports suggest symptom improvement may occur. Should be prioritized or stratified in early prospective studies.
Breast cancer	Usually moderate to high	Possible relevance in selected patients, depending on subtype, extent of peritoneal involvement, systemic options, and prognosis. Could be included with stratification by subtype and prior systemic therapy.
Gastric cancer and colorectal cancer	Moderate to low	MA is common, but radiosensitivity is less predictable.
Pancreatic adenocarcinoma	Low to moderate	MA is frequent in advanced disease, but limited radiosensitivity may reduce the expected benefit.
Peritoneal mesothelioma	Variable	Potential role uncertain; peritoneal distribution may be suitable anatomically, but evidence for ascites-directed WPRT is lacking.
Sarcomas/desmoplastic small round cell tumour	Variable	Should be interpreted separately from common epithelial causes of MA.

At the same time, there is a broader clinical rationale for radiotherapy as a symptom-directed intervention even when durable oncologic control is not the primary goal. Radiotherapy is already widely used in symptomatic treatment to relieve pain, bleeding, and compressive symptoms. By analogy, WPRT may be conceptually relevant in MA, where the primary target would be a distressing symptom complex associated with peritoneal tumor activity. This may be especially relevant in patients with recurrent MA in whom standard symptomatic measures provide only transient relief and systemic options are limited, ineffective, or no longer feasible. However, such reasoning should not be interpreted as evidence of efficacy. Rather, it underscores why the topic warrants prospective evaluation.

## Existing evidence relevant to WPRT in malignant ascites

To date, no dedicated studies have investigated radiotherapy specifically as a symptomatic treatment for MA. The available evidence is indirect and derives from studies of whole-abdominal or abdominopelvic radiotherapy for diseases associated with peritoneal involvement. Most of these reports focused on conventional oncological endpoints such as response, progression, survival, or general symptom control rather than on MA itself.

Cancers most commonly associated with peritoneal infiltration and MA include ovarian, breast, colorectal, and pancreatic cancers. Less commonly, MA may occur in lung cancer, lymphomas, and peritoneal mesothelioma. In many of these diseases, radiotherapy has been used in selected situations, either as part of definitive, adjuvant, or palliative treatment [14].

It is reasonable to consider that WPRT might be more effective in patients with relatively radiosensitive malignancies [14]. Nevertheless, this remains an extrapolation rather than a conclusion supported by direct data.

Abdominal radiation therapy has been explored in ovarian cancer, endometrial cancer, gastric cancer, desmoplastic small round cell tumors, peritoneal sarcomas, colorectal metastases, and peritoneal carcinomatosis, in both adjuvant and palliative settings, and either alone or combined with systemic treatment [15], [16], [17], [18], [19], [20], [21].

Because existing data are heterogeneous and do not directly address WPRT as a dedicated symptomatic treatment for MA, the main reports are summarized in Table 3 to clarify the tumor settings, treatment approaches, reported outcomes, and limitations of the evidence.

**Table 3:** Indirect clinical evidence relevant to large-field abdominal or abdominopelvic radiotherapy in malignancies with peritoneal involvement.

Study [reference]	Tumor and settings	Number of patients	Field, technique, dose, fractionation	General outcomes	MA-relevant outcomes	Toxicity	Key limitations, comments
Hepp et al. 2002 [15]	Ovarian cancer, adjuvant	60	WART, 22 Gy in 22 fractions, in 90 % and 25 Gy in 25 fractions in 10 % of patients. A boost to the pelvis to reach 45 Gy at 1.8 cGy/fraction.	Oncological outcomes. OS 55 % at 5 years (median follow-up 96.5 months). The abdominal control rate was 83 %.	Not reported	Vomiting and diarrhea in 20 %, with no stratification as to the reason (RTH/CHTH), good abdominal tolerance reported overall	Indirect relevance. 19 patients of 60 received platinum-based chth. Retrospective, historical study (1985–1998).
Sutton et al. 2005 [16]	Endometrial cancer, adjuvant	174 assessed for radiation toxicity	WART (30 Gy in 20 fractions) with subsequent boost to the pelvis 19 8 Gy at 180 cGy per fraction. For positive para-aortic nodes, an additional boost of 1.5 Gy for a total dose of 4.5 Gy was applied.	Oncological outcomes. Recurrence-free survival rates 29% and 27 % at 3 years for the typical endometrial adenocarcinoma and high-risk histologies, the survival rates 31 % and 35 %, respectively.	Not reported	Hematologic: G3 11 %, G4 2 %. GI (nausea, diarrhea): G3 11 %, G4 4 %. Hepatic toxicity G3 2 %, G4 1 %. Two patients developed post-RTH ascites.	Indirect relevance due to adjuvant settings
Randal et al. 1995 [17]	Endometrial cancer, comparison of WART vs. chemotherapy	396	WART: 30 Gy/20 fractions + boost 15 Gy	Oncological outcomes. 5-year survival 55 % in chth, 42 % in WART.	Not reported	Treatment-related deaths: 8 (4 %) for chth, 5 (2 %) for WART.	Indirect relevance. The comparator was systemic therapy.
Orhan et al. 2011 [18]	Locally advanced gastric cancer	90	WART (45 patients; 20 Gy whole abdomen + 25.2 Gy on tumor/lymph nodes) vs. standard field RTH (45 patients, 45 Gy) + adjuvant chth	Oncological outcomes.	Not reported.	RTH used with adjuvant chth. GI G3/G4 toxicities (mainly diarrhea) higher for WART (16 % vs. 4 %, p=0.049).	Indirect relevance. Combined treatment modality setting.
Pahuja and Mundodan 2018 [19]	Abdominal desmoplastic small round cell tumor	1 (case report)	Post-surgery WAPRT (IMRT) + chth	Oncological outcomes.	Mild ascites at admission. 22 months later, disease-free.	G1 nausea and vomiting. No haematological toxicity.	Indirect relevance. Radiosensitive tumor. Combined treatment modality setting.
Honoré et al. 2015 [20]	Abdominal desmoplastic small round cell tumour; multimodal therapy. Adjuvant RTH.	7 patients	Post-surgery WAPRT	Oncological outcomes.	Not reported	Limited data, typical	Indirect relevance, RTH was part of multimodal treatment, rare tumor.
Reiss et al. 2021 [21]	Advanced solid malignancies with peritoneal carcinomatosis. Palliative setting.	22 patients with solid tumors (including 8 with ovarian/fallopian tube cancer).	Low-dose fractionated WART (21.6 Gy in 36 fractions) combined with veliparib	Oncological – PFS, OS.	Not reported	WART-specific toxicity not evaluable (multimodal treatment).	Phase I design; RTH combined with radiosensitizer; heterogeneous tumor types; not MA-directed. Patients with uncontrolled MA were excluded.
De Meerleer et al. 2011 [22]	Chemotherapy-resistant ovarian cancer with bulky peritoneal disease. Palliative setting.	13 heavily pretreated ovarian cancer patients with bulky peritoneal disease. Palliative setting. Dose up to 33 Gy (8 patients)	WAPRT (IMAT),	In 9 patients completing treatment, complete symptom alleviation was reported except for ascites (partial response).	Partial MA response – paracentesis was reduced by 50 % in a single patient, who received WAPRT specifically due to MA.	G3 diarrhea in 2 patients, G3 nausea in 1 patient. Acute postrenal failure due to obstruction by a tumor mass in 1 patient who underwent additional pelvic RT afterwards.	A single-institution limited series. A minimal Karnofsky performance score of 50. WAPRT resolved intestinal obstruction for a substantial period.
Tinger et al. 2001 [23]	Advanced or recurrent ovarian carcinoma. Palliative setting.	62 patients received RTH to the abdomen or pelvis, or both.	12 received WAPRT, 12 WAPRT with boosts to the paraaortic lymph nodes/medial diaphragms/pelvis. Total doses for whole abdomen/pelvis were 4.5–57.6 Gy (median 42 Gy).	High response rates in terms of pain, mass, bleeding, skin involvement, obstruction.	8 patients treated specifically for MA, 25 % experienced relief.	5 % developed G3/4 nausea, vomiting or myelosupression	Retrospective, small MA subgroup, ovarian cancer specific, limited details on MA response.
Ishibashi et al. 2020 [24]	Pleural or peritoneal disseminated tumors (e.g., colorectal, ovarian). Palliative setting.	13 patients with peritoneal dissemination + 2 with peritoneal + umbilicus.	Total doses between 27 in 9 fractions to 56 Gy in 28 fractions. Detailed data uncertain.	Used for pain relief or hemostatic intent.	No conclusions regarding MA.	No G2 or higher RT-related toxicities were observed.	Small sample; symptom endpoints other than MA.
Lee et al. 2003 [25]	Endometrial adenocarcinoma with peritoneal dissemination. Palliative setting.	11 patients with residual disease for adjuvant WART.	WART, 1.0–1.5 Gy fractions to 30 Gy with optional boosts on pelvis/paraaortic lymph nodes.	RT eliminated small intraperitoneal deposits	No data concerning MA.	Lack of detailed data. Radiation hepatitis in one patient 15 months after RT.	Not MA-directed; endometrial cancer specific. Historical study (1988–1999).

WART, whole abdominal radiotherapy; WAPRT, whole abdominopelvic radiotherapy; IMRT, intensity-modulated radiotherapy; IMAT, intensity-modulated arc therapy.

The available clinical literature provides only indirect and highly limited support for considering external beam radiotherapy in the setting of MA. De Meerleer et al. treated 13 patients with chemotherapy-resistant ovarian cancer and extensive peritoneal involvement using arc-based whole abdominopelvic irradiation. Among patients able to complete the planned course, global symptom relief was reported, whereas ascites improved partially [22]. In the retrospective series by Tinger et al., radiotherapy was delivered to patients with advanced or recurrent ovarian carcinoma, but MA was the primary treatment indication in only 8 of 80 cases. Therefore, although responses were described as favorable, this subgroup is insufficient to define the effect of radiotherapy on ascites control [23]. Other reports are even less directly applicable. Ishibashi et al. included patients with peritoneal dissemination, but treatment was directed toward pain palliation or bleeding control rather than MA itself [24]. Lee et al. reported clearance of small intraperitoneal tumor deposits after whole abdomen radiotherapy in disseminated endometrial adenocarcinoma; however, ascites was not the focus of that study, and its findings cannot be taken as evidence for MA-directed radiotherapy [25].

## Timing and patient selection considerations

An important and unresolved issue is when WPRT should be considered. One possibility is that patients with mild or moderate ascites and evidence of progressing peritoneal involvement could benefit from earlier intervention before the ascites causes significant suffering. Another option is that treatment should be reserved for patients with recurrent, clinically significant ascites after failure of standard measures.

The feasibility and potential usefulness of WPRT would depend heavily on performance status, expected survival time, pace of fluid reaccumulation, prior therapies, and patient preference. Primary tumor type and expected radiosensitivity should also be considered, as they may affect the likelihood and durability of symptomatic benefit from WPRT.

Patel et al. suggested that radiotherapy delivered in the last month of life may provide little benefit overall [26]. However, the systematic review by Park et al. indicated that, even among patients who survived for less than 4 weeks, approximately 1 in 4 experienced symptomatic relief after palliative radiotherapy, particularly with shorter fractionation schedules [27]. These findings support individualized decision-making rather than rigid exclusion based solely on prognosis.

The biological effects of radiotherapy may also extend beyond simple tumor shrinkage. It seems conceivable that effective symptom control of MA could, in some patients, improve oral intake, mobility, or overall condition. Any impact on cachexia would likely depend on the broader clinical context, as the relationship between cancer, treatment, malnutrition, cachexia, and sarcopenia is complex [28]. At present, however, these potential indirect benefits remain speculative, and there are no data defining optimal timing, eligibility criteria, or prognostic factors for WPRT in MA.

Because WPRT for MA remains investigational, any suggested selection criteria should be regarded as a starting point for prospective protocol development rather than as recommendations for routine clinical practice. Nevertheless, specifying a preliminary eligibility framework may facilitate the design of early phase studies and improve comparability across future reports. A preliminary trial design framework for future studies is presented in Table 4.

**Table 4:** Preliminary eligibility framework for WPRT studies in malignant ascites.

Domain	Suggested criterion for early WPRT trials in MA
Performance status	ECOG 0–2 preferred; ECOG ≥3 only in predefined exceptional cases, particularly stable non-cancer-related disability, if MA is a major symptom driver and treatment is feasible
Expected survival	Preferably ≥8–12 weeks; generally exclude patients with expected survival <4 weeks
Histology/radiosensitivity	Stratify by primary tumor type and expected radiosensitivity, e.g., lymphoma, ovarian/fallopian tube/primary peritoneal carcinoma, endometrial or cervical cancer, breast cancer, gastric cancer, colorectal cancer, and pancreaticobiliary tumors; poorly radiosensitive histologies should be analyzed separately or included only with clear justification
Ascites burden and stability	Symptomatic recurrent MA, e.g., repeated paracentesis, clinically relevant reaccumulation, or ≥2 drainages within 6 weeks; tense/massive ascites should be drained and fluid/electrolyte instability corrected before WPRT
Radiotherapy safety	Review prior abdominal/pelvic irradiation and organ-at-risk constraints; exclude patients if safe constraints cannot be met
Prior surgery/abdominal complications	Exclude major abdominal surgery within 4 weeks, unhealed wounds, active fistula, bowel perforation, uncontrolled obstruction, or severe postoperative complications
Timing of WPRT	Consider after recurrent symptomatic MA is established, but before irreversible functional decline, severe cachexia, or terminal deterioration
Ascites volume tresholds	Symptomatic, measurable, or recurrent MA; no fixed volume thresholds proposed. Record baseline ascites volume, drainage frequency, re-accumulation time, symptoms, and paracentesis need. Near-daily drainage or clinical instability should be considered cautionary/exclusion features

## Technical feasibility and treatment planning considerations

### Anatomical and imaging challenges

The peritoneal cavity is enclosed by the peritoneum, which includes the parietal peritoneum along the abdominal wall and the visceral peritoneum covering abdominal organs [29]. Relevant structures include the mesentery, mesocolon, and the greater and lesser omentum, as well as multiple peritoneal recesses. From the perspective of computed tomography (CT) and magnetic resonance imaging (MRI), the peritoneal cavity is not delineated as a discrete organ. Instead, its boundaries are inferred from anatomical relationships and from identification of intraperitoneal vs. extraperitoneal structures.

The complex anatomy of the peritoneal space and the often subtle nature of peritoneal involvement restrict CT’s sensitivity. Even extensive infiltration may be radiologically occult. MRI is generally more sensitive, and positron emission tomography (PET) may provide additional information in selected cases. In practice, assessment of peritoneal involvement often combines imaging with other data, including:

– prior surgical findings, such as aborted operations due to diffuse peritoneal disease,
– cytological evaluation of PF,
– rising tumor markers,
– the presence of ascites not explained by liver dysfunction,
– radiologic and clinical manifestations in patients with massive peritoneal and/or integument and body wall infiltration or peritoneal-cutaneous fistula formation [30], 31].

### Planning and logistics

Established standards settle many general issues, such as identifying critical organs, respecting tolerance doses, and selecting an irradiation technique. However, WPRT for MA raises additional questions:

– whether MRI or PET should be used when CT findings are equivocal,
– whether patients with suspected MA require further diagnostic work-up to exclude alternative causes of ascites,
– whether an additional boost to apparent areas of peritoneal involvement would improve effectiveness in regard to MA,
– whether incomplete coverage of diffuse peritoneal involvement might still be sufficient to reduce PF production.

Accurate radiation delivery to the peritoneal cavity cannot be reliably planned in the presence of significant PF. Therefore, WPRT would likely require close coordination between drainage and treatment planning. In rapidly reaccumulating MA, the interval between paracentesis, planning CT, and treatment delivery should be minimized.

The key steps would include:

– therapeutic paracentesis, potentially with ultrasound confirmation of maximal evacuation,
– planning CT performed immediately after drainage,
– rapid treatment planning by the radiation oncology and physics team,
– timely delivery of WPRT before significant fluid reaccumulation occurs.

After large-volume paracentesis, intravenous albumin supplementation may be beneficial in selected patients.

### Dose and fractionation

Because the primary objective is symptom relief, shorter, simpler regimens may be preferable in palliative care. In other palliative settings, such as painful bone metastases or tumor-related bleeding, prolonged fractionation schedules usually do not provide significant advantages over shorter ones [32], [33], [34], [35], [36], [37]. By analogy, concise WPRT regimens may be reasonable to investigate first in MA.

A single-fraction strategy could be attractive due to its minimal treatment burden and logistical ease. However, the optimal dose-fractionation remains unknown. In addition, some tumor types may be relatively insensitive to conventional fractionation, whereas hypofractionation may offer theoretical advantages in selected contexts. These issues remain entirely unresolved for MA.

### Illustrative planning example

Figure 1 illustrates a representative radiotherapy planning simulation for WPRT. The example is intended to demonstrate the technical planning concept of external-beam irradiation of the peritoneal compartment, including dose distribution and organ-at-risk sparing considerations. It should not be interpreted as clinical evidence of efficacy, safety, or generalizable feasibility. Rather, it provides a dosimetric illustration of how WPRT might be technically planned in a selected scenario, while underscoring the need for prospective evaluation before any clinical implementation. Table 5 presents planning and dosimetric characteristics of the illustrative WPRT simulation.

**Figure 1:**
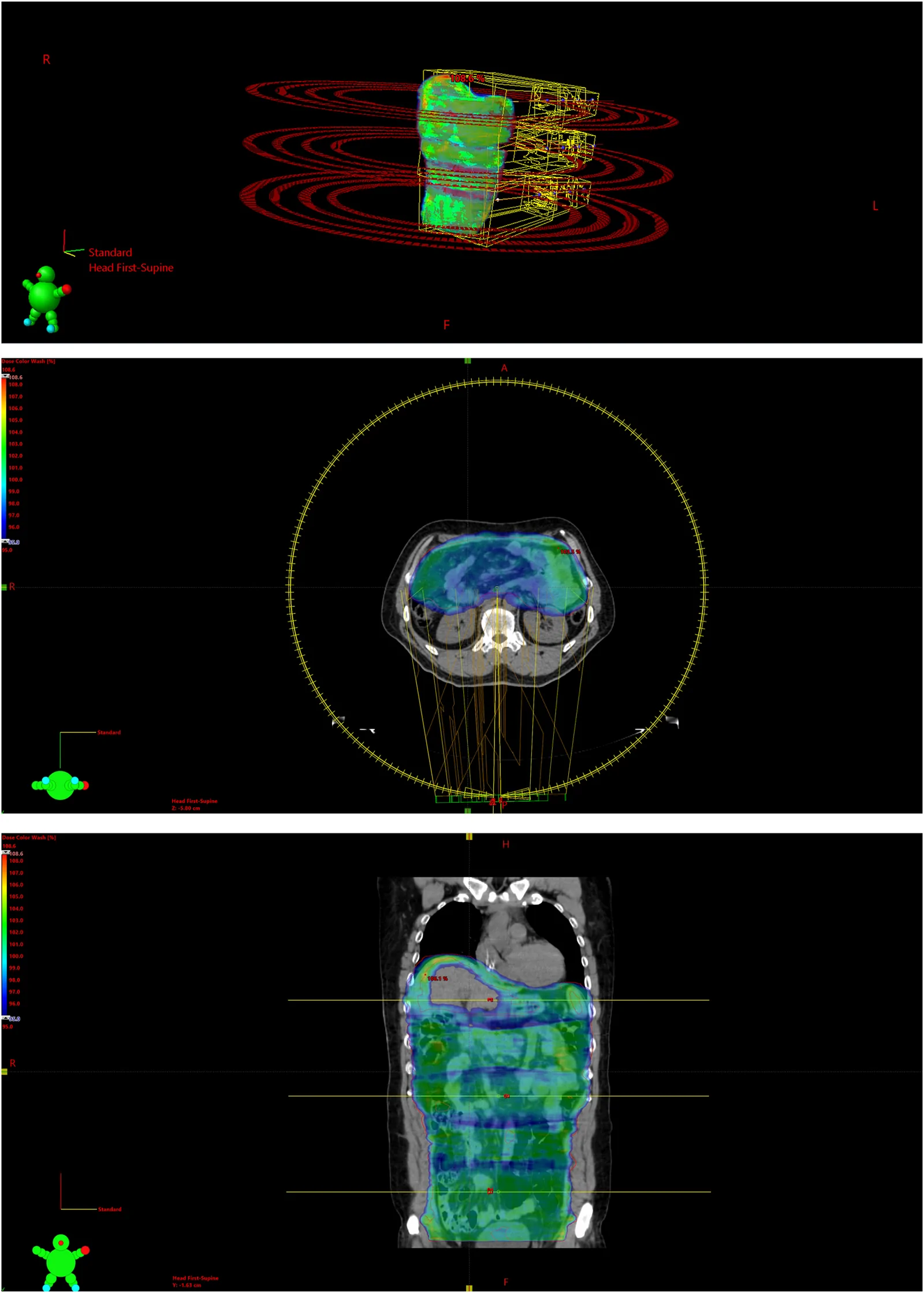

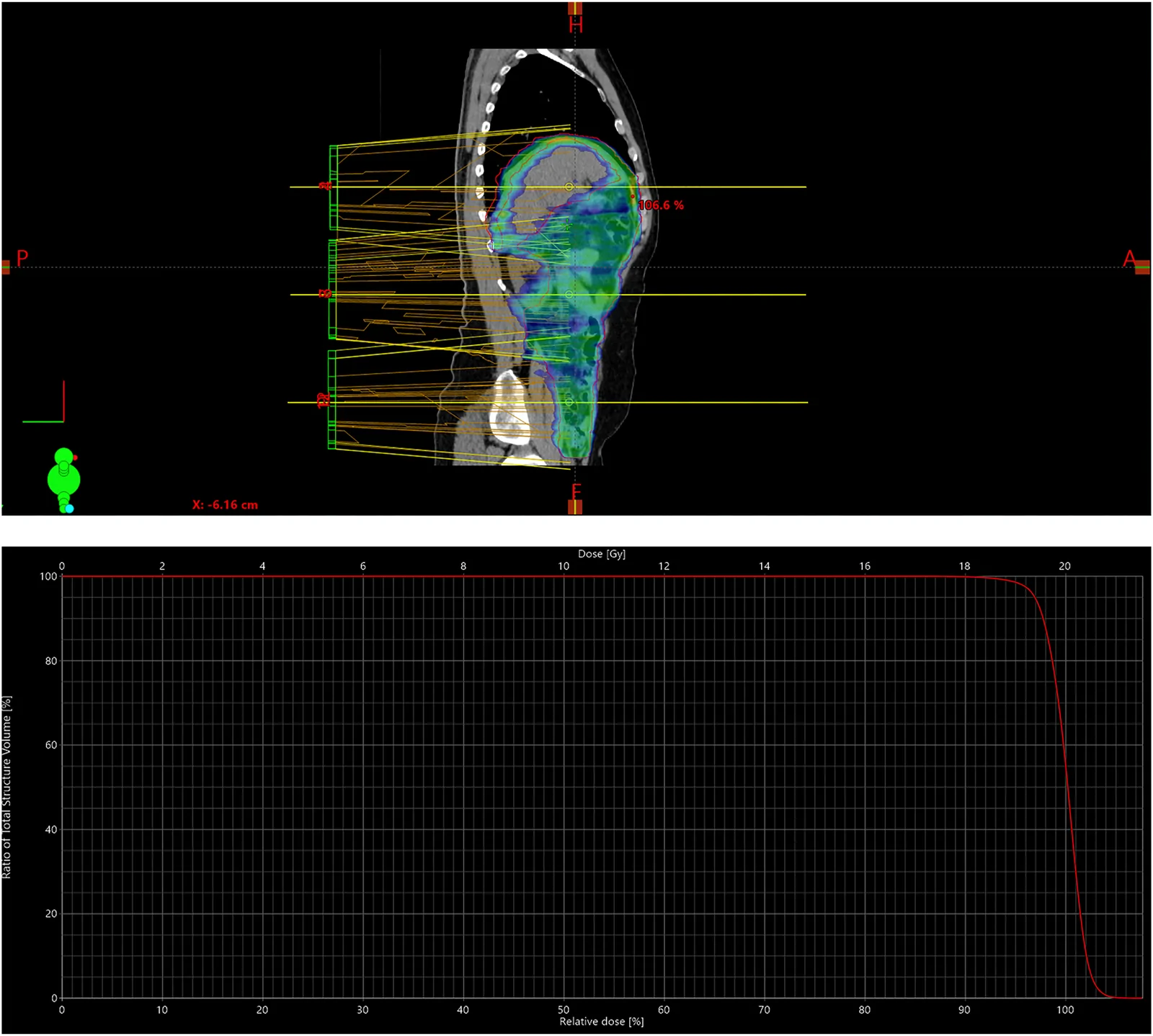
Illustrative VMAT (Volumetric Modulated Arc Therapy) whole peritoneal radiotherapy dose distribution using a single-fraction 8 Gy prescription. This example is intended only to visualize a possible planning concept and should not be interpreted as evidence of clinical feasibility, safety, or efficacy. Detailed contouring rules, organ-at-risk constraints, and dose-volume parameters require prospective protocol definition.

**Table 5:** Planning and dosimetric characteristics of the illustrative WPRT simulation.

Category	Suggested content
Planning technique	VMAT
Simulation	CT-based planning, slice thickness: 3 mm, patient position: HFS
Target	Peritoneal surfaces (CTV)
Margins	CTV–PTV margin: 5 mm
Dose prescription	Total dose: 8 Gy in 1 fraction
Coverage	PTV: V95 %=98.2 %, Dmax – 108.6 %
Organs at risk	bowel: Dmax <31 Gy stomach: Dmax <22 Gy liver: dose for volume of organ V700 cc <11,6 Gy kidneys: V200 cc <9,5 Gy spinal cord: Dmax <14 Gy

VMAT, Volumetric Modulated Arc Therapy; CT, computed tomography; HFS, head first supine; CTV, clinical target volume; PTV, planning target volume; OAR, organs at risk; DVH, dose volume histogram; Dmax, maximum dose tolerance.

### Safety considerations and limitations

The toxicity profile of abdominal radiotherapy is well recognized. Potential adverse effects include abdominal pain, radiation enteritis, nausea, vomiting, diarrhea, constipation, mucosal inflammation, intestinal perforation, and chronic pancreatitis [38], [39], [40]. In longer-term survivors, abdominal radiotherapy may also contribute to metabolic complications such as insulin resistance, metabolic syndrome, and type 2 diabetes [41], 42]. In the palliative MA population, however, late metabolic effects may be less clinically relevant because of limited survival.

Safety must also be considered in relation to patient frailty. Radiotherapy is usually best tolerated in patients with relatively preserved general condition, but palliative practice often extends the treatment schedule to selected patients with ECOG 3 or even 4 if the goal is symptom reduction or control of distressing or life-threatening manifestations. This principle is already accepted in other palliative radiotherapy indications, including pain, bleeding, and pressure symptoms [27], 43]. The same reasoning may apply to MA in selected cases, provided that the expected benefits outweigh the burden of treatment and that informed consent is obtained following a realistic discussion.

The possibility of treatment-related worsening of ascites should also be acknowledged. Barrera et al. described a case of post-radiotherapy chylous ascites in a patient treated for gastric cancer, ultimately attributed to congenital absence of the cisterna chyli identified by lymphangiography [44]. Such reports are extremely rare and do not negate the rationale for WPRT, but they highlight the need for caution when discussing expected benefits and risks.

Several broader limitations should be emphasized:

– Patients with MA often have multimorbidity, cachexia, and limited life expectancy,
– Prior surgery, systemic treatment or irradiation may increase toxicity risk,
– No evidence currently defines the safest or most effective WPRT protocol in this setting.

A limited body of evidence may help establish an optimal protocol. For example, Desai et al. reported reduced toxicity with intensity-modulated radiation therapy (IMRT) for desmoplastic small round cell tumor compared with conventional 2-dimensional radiation therapy [45].

For the above-mentioned reasons, if WPRT is explored clinically, simple and minimally burdensome schedules appear most appropriate at the initial stage.

### Dose, fractionation, and toxicity considerations

The optimal dose and fractionation for WPRT in MA are unknown. Although 8 Gy in a single fraction is conceptually attractive because of its low treatment burden and use in other palliative radiotherapy settings, this analogy is imperfect. WPRT would involve large abdominal target volumes and exposure of bowel, liver, kidneys, and bone marrow. Thus, 8 Gy should be regarded only as a hypothetical low-burden regimen, not an established or preferred approach.

Alternative schedules could include 20 Gy in 5 fractions or 30 Gy in 10 fractions, but higher total doses and longer treatment courses may increase gastrointestinal, hematologic, renal, and hepatic toxicity, particularly in frail patients with cachexia, malnutrition, dehydration, impaired organ function, prior systemic therapy, or limited marrow reserve. Expected toxicities include nausea, vomiting, diarrhea, abdominal pain, anorexia, fatigue, dehydration, cytopenias, and possible renal or hepatic deterioration. Bowel ulceration, obstruction, bleeding, or perforation are important theoretical risks, especially with bowel wall involvement or impending perforation.

Therefore, any WPRT regimen should be considered investigational and should require prospective evaluation with predefined organ-at-risk constraints, supportive care protocols, toxicity monitoring, and early stopping rules.

## Conceptual patient selection and clinical caution criteria

Because WPRT for MA remains conceptual and hypothesis-generating, no validated eligibility criteria exist. From a safety perspective, WPRT would be least appropriate for patients with very short expected survival, poor performance status, rapid clinical deterioration, bowel obstruction or ileus, suspected impending perforation, extensive bowel wall involvement, active gastrointestinal bleeding, uncontrolled infection or sepsis, severe cachexia or malnutrition, major renal/hepatic dysfunction, inadequate marrow reserve, prior extensive abdominal or pelvic radiotherapy, or very rapid ascites re-accumulation after drainage. In these situations, established symptom-directed measures such as paracentesis, drainage, infection control, analgesia, nutritional/supportive care, and systemic therapy when appropriate should take priority.

If investigated prospectively, WPRT would most plausibly be limited to carefully selected patients with symptomatic MA, reasonable performance status, sufficient expected survival for treatment and response assessment, controlled infection status, acceptable organ and marrow function, no bowel obstruction or high-risk bowel features, and no prohibitive prior abdominal irradiation.

## Evidence gaps and future research priorities

The main gap in the literature is straightforward: no study has directly evaluated WPRT as a dedicated symptom-directed treatment for MA. As a result, nearly every clinically relevant question remains open, including:

– which primary tumors are most suitable for this approach,
– what degree and pattern of peritoneal involvement are required,
– when treatment should be offered during the course of the disease,
– what dose and fractionation are optimal,
– whether complete peritoneal coverage is necessary,
– whether radiologically visible disease should receive an additional boost,
– how previous abdominal treatment affects safety and efficacy,
– which outcomes best reflect meaningful clinical benefit.

Future studies should focus on patient-centered endpoints. Primary outcomes should include changes in quality of life, assessed with validated instruments, and direct assessment of ascites-related symptom burden, including abdominal distension, pain, dyspnea, nausea, and the ability to maintain oral intake. Secondary outcomes should include:

– frequency and cumulative volume of therapeutic paracenteses,
– time to next paracentesis,
– patient-reported need for drainage,
– nutritional status,
– treatment-related toxicity, especially gastrointestinal adverse effects,
– overall survival.

The most rigorous design would be a prospective randomized controlled trial comparing WPRT plus best supportive care (BSC) with BSC alone in patients with recurrent MA. Blinding does not appear to be essential in this setting. At the earliest stage, a pilot prospective study with carefully matched historical controls could help establish feasibility, safety, and early signals of efficacy.

## Potential clinical implications

If future studies confirm the benefit, WPRT could potentially offer several advantages in selected patients with recurrent MA, including:

– reduction of ascites-related symptom burden,
– decreased need for repeated paracentesis or permanent drainage systems,
– improvement in quality of life,
– possible stabilization of nutritional status by reducing recurrent fluid loss and treatment-related deproteinization,
– better pain control in selected cases with symptomatic peritoneal disease,
– improvement in general condition in a subset of patients.

Such effects might in some cases be sufficient to allow continuation or reintroduction of systemic therapy, although this possibility remains speculative.

An additional theoretical consideration concerns paraneoplastic hyponatremia, which is common in cancer patients and is associated with impaired quality of life and worse outcomes [46], 47]. Experimental data suggest that low extracellular sodium may influence tumor cell proliferation and invasiveness [48], 49]. In patients with peritoneal cancerous involvement, cytoreductive effects of radiotherapy might conceivably contribute to transient improvement in this metabolic disturbance. However, this concept is hypothetical and unsupported by direct evidence in the context of WPRT and MA.

Figure 2 presents a hypothesis-generating conceptual flowchart for a prospective evaluation of WPRT in MA. It does not represent a clinical management algorithm or an established treatment protocol. Symptom-directed management remains the priority, whereas WPRT, if considered, should be limited to a prospective investigational framework with careful eligibility assessment, predefined safety constraints, and clinically meaningful endpoints.

**Figure 2:**
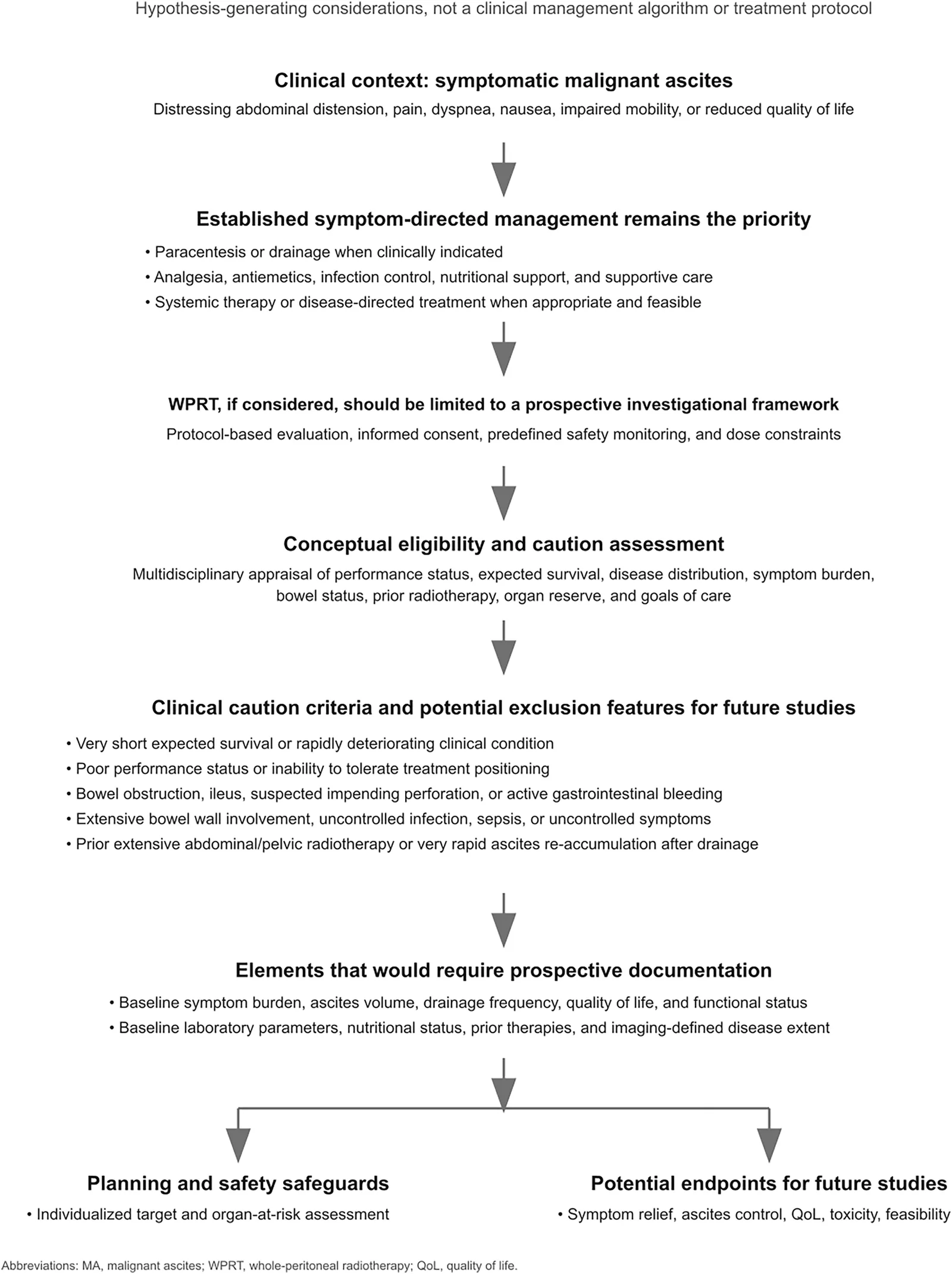
A hypothesis-generating conceptual flowchart for a prospective evaluation of WPRT in MA. It does not represent a clinical management algorithm or an established treatment protocol. Symptom-directed management remains the priority, whereas WPRT, if considered, should be limited to a prospective investigational framework with careful eligibility assessment, predefined safety constraints, and clinically meaningful endpoints.

If WPRT in the context of MA proves effective, it would represent a symptom-oriented use of radiotherapy, analogous to established palliative indications such as analgesic, hemostatic, or decompressive treatment. Its potential applicability across multiple cancer types and healthcare settings would further support its relevance. Palliative radiotherapy already constitutes a substantial part of radiotherapy practice and is regarded as efficient and cost-effective [50].

## Conclusions

MA remains a major problem in palliative care. Although no studies have directly assessed WPRT as a dedicated symptom-directed treatment for MA, the available literature provides a biologically plausible and clinically relevant rationale for further investigation. Existing evidence is indirect, heterogeneous, and insufficient to define patient selection criteria, optimal dose-fractionation, expected magnitude of benefit, or safety profile in this setting. At present, WPRT should be regarded as an investigational palliative approach rather than an established treatment option. Nevertheless, modern radiotherapy techniques, the limitations of current MA management, and the symptom-oriented logic of palliative irradiation together support prospective evaluation. Future studies should determine whether WPRT can reduce ascites-related symptom burden, improve quality of life, and decrease the need for repeated drainage procedures in selected patients.

We hope that our narrative review will stimulate discussion and clinical research among radiation oncologists, oncologists, palliative care physicians, and other professionals regarding the potential use of WPRT as a palliative symptomatic treatment for malignant ascites.
